# Prenatal diagnosis of multiple large subchorionic placental cysts with intracystic hemorraghe

**Published:** 2017-12

**Authors:** I Witters, P Sieprath, C Van Holsbeke, C Theyskens, K Deraedt

**Affiliations:** Department of Obstetrics and Gynecology, Ziekenhuis Oost-Limburg, Genk (ZOL), Genk, Belgium; Neonatology, Ziekenhuis Oost-Limburg, Genk (ZOL), Genk, Belgium; Department of Pathology, Ziekenhuis Oost-Limburg, Genk (ZOL), Genk, Belgium

**Keywords:** intrauterine growth restriction, placental cyst, pregnancy, ultrasound

## Abstract

Subchorionic placental cysts occur in up to 5% of pregnancies. Large and numerous placental cysts increase the risk for intrauterine growth restriction. We describe a case with large multiple subchorionic placental cysts complicated by intracystic hemorraghe and fetal growth restriction.

## Introduction

Subchorionic placental cysts occur in up to 5 % of pregnancies.

Small single subchorionic placental cysts are of no clinical importance and in these cases obstetrical management should not be altered, but large cysts and multiple cysts can be associated with intrauterine growth restriction. Therefore in these cases fetal growth evaluation especially in the third trimester is indicated and obstetrical management is determined by fetal growth.

We report for the first time a case of multiple enormous subchorionic placental cysts each with intracystic hemorraghe followed by preterm delivery at 34 weeks for intrauterine growth restriction.

## Case report

A 37-year-old gravida 2 para 1 (one previous c-section for breech position with a daughter of 3300 g) presented at 19 weeks due to an increased alfafetoprotein on an integrated biochemical screening test (AFP: 3.5 MoM).

Ultrasound revealed normal fetal growth, no structural anomalies, normal dopplers. Placental lakes (>50 % of the placenta) were present. A non- invasive prenatal test (NIPT) on parental request was normal.

**Figure 1:**
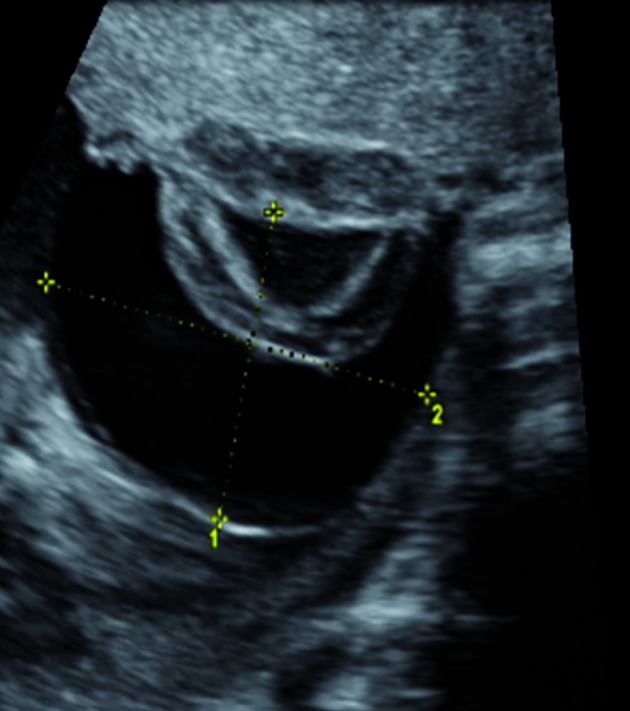
— Ultrasound of one of the cysts, measuring 5 cm, located at the fetal placental side and containing echogenic material suggestive of hemorraghe.

Follow-up scan for growth at 26 weeks revealed normal fetal growth, normal dopplers and 2 subchorionic placental cysts located near the umbilical cord insertion, measuring 5 x 3 and 5x 4 cm (Fig. 1).

Gestational diabetes, diagnosed following abnormal glucose challenge test and OGTT, was treated with dietary advise.

At 30 weeks fetal growth had declined (percentile 11), amniotic fluid and fetal dopplers were normal, but the amniotic fluid (AF) was stained and fetal movements were decreased. The placental cysts were equal in size and two intraplacental echogenic cystic lesions were reported.

The glucose profile showed postprandial sub- optimal low glycemic values and patient received dietary advise with improvement of glycemia and of fetal movements.

**Figure 2:**
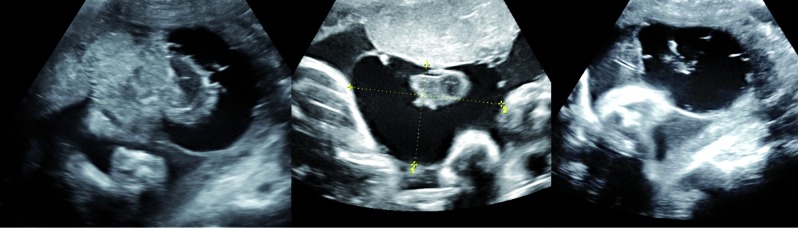
— Ultrasound at 32 weeks with multiple large (5cm) cysts with intracystic hemorraghe.

At 32 weeks ultrasound revealed 6 large subchorionic placental cysts, all measuring > 5 cm, with intracystic heterogenous material compatible with clotting (Fig. 2). Fetal growth was on the 9 th centile with stained amniotic fluid and normal fetal dopplers.

Due to the sudden change of the placental aspect the patient was admitted for fetal monitoring and a repeat c-section was at 34 weeks for suboptimal fetal monitoring. A girl of 1850 g with Apgar scores 6 and 8 at 1 and 5 minutes and arterial cord PH of 7.21 was born, with an uncomplicated neonatal course.

**Figure 3:**
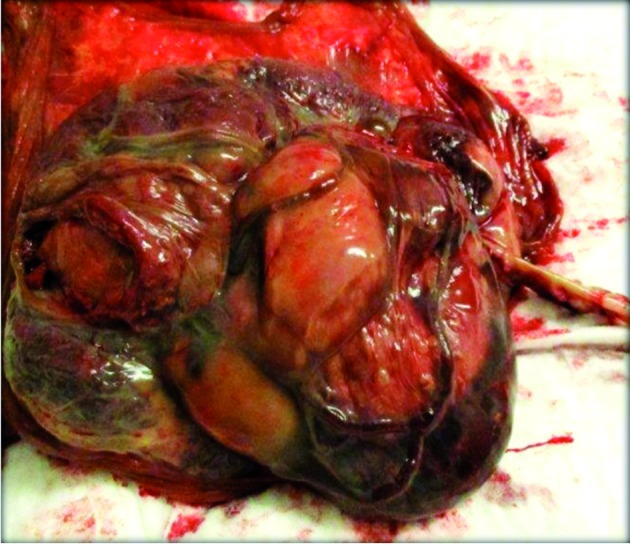
— macroscopic view on the fetal side of the placenta with large multiple placental cysts.

The placenta weighed 513 g, measured 16x 15 x 6 cm and had a 50 cm 3-vessel cord. Multiple large subchorionic cysts (measuring each 6 cm), all with intracystic hemorraghe and massive perivillous fibrin deposits were described (Fig.3).

## Discussion

Cystic masses on the fetal side of the placental surface have been referred to by ‘chorionic cysts’, ‘subchorionic cysts’ and ‘membranous cysts’.The overall incidence of such placental cysts is up to 5 % of pregnancies (Brown et al., 2002). Often these cysts are located near the umbilical cord insertion and can be already present in the first trimester. By ultrasound these cysts occur as echo-free cavities under the fetal plate without color Doppler signal in the lesion. The differential diagnosis includes subchorionic and subamniotic hematoma (with hypoechoic content or with a more solid content inside the cyst), chorioangioma (with color Doppler signal), placental lakes and cord cysts.

As in this report, subchorionic cysts have been described to be associated with perivillous fibrin deposition. Trofoblastcells outside the villi (X cells) are also found in the wall of the cysts. It has been suggested that cystic formation occurs in subchorionic fibrin deposits. The location near the umbilical cord insertion could be related to fetal traction on the fixed cord portion, with weakening of the nearby chorionic surface (Brown et al., 2002). Regarding its clinical importance Brown et al reviewed 34 cases of placental surface cysts and found that small simple cysts (<2 cm) are not likely of any clinical significance but cyst size >4.5 cm was associated with 33% risk of growth restriction (FGR). Multiple cysts (>3) occur infrequently (only 2/34 cases in the review) and were uniformly associated with FGR (Brown et al., 2002; Raga et al., 1996). Placental cysts located near the placental cord insertion could also influence with fetal growth by interfering with the umbilical cord circulation |(Raga et al., 1996).

For small cysts, no alteration of obstetrical management is indicated. For larger and also for multiple cysts serial ultrasound examinations, especially during the third trimester, are important to detect intracystic hemorrhage and FGR. The latter cases with FGR should be managed by following the fetal growth restriction protocol. As in the present case a decision for hospital admission for fetal surveillance was not made based on fetal growth but on the sudden ultrasonographic change in growth of the placental cysts with intracystic hemorrhage. The mode of delivery is mainly dependent on the fetal condition and on the risk of cyst rupture. In a review of six previously reported cases of subchorionic cysts, three resulted in spontaneous vaginal deliveries (cyst sizes 5-6 cm) and three in caesarian sections because of a huge cyst (12 cm), FGR with nonreactive nonstress test and FGR, oligohydramnios and transverse lie (Hong et al., 2007).

In the present case at first a high second trimester alfafetoprotein (AFP) was the reason for referral. We do not screen for AFP in our centre but high levels of AFP are a risk factor for poor pregnancy outcome including, preterm birth, pregnancy- induced hypertension, fetal death, low birth weight and low APGAR scores (Puntachai et al., 2015).

Massive perivillous fibrin deposition, as in the placental examination of this case, is almost always accompanied by FGR and an increased risk for fetal demise (Devisme et al., 2017).

At 26 weeks 2 echogenic cystic lesions were noted, known to be associated with intervillous fibrin deposits and thrombosis (Proctor et al., 2010; Devisme et al., 2017).

In the third trimester multiple very large subchorionic cysts appeared, suggesting cyst formation in fibrin deposits. The size and number of cysts were infavorable for fetal outcome.

Only one previous report (Hong et al., 2007) describes intracystic hemorraghe in a large subchorionic cyst, as was also seen in our case. In the present case the extend, size and number of cysts are very unusual.

## Conclusion

We report an unusual case of multiple large subchorionic placental cysts with intracystic hemorraghe associated with FGR. The presence of massive perivillous fibrin deposits favours previously described cystic formation in subchorionic fibrin deposits. As for the clinical management, cyst size and number of cysts are determinants for the risk of fetal growth restriction and unfavorable pregnancy outcome.

## Declaration

The authors report no financial or commercial conflicts of interest.
